# Improved safety and effectiveness of imaging predicted for MR mammography

**DOI:** 10.1038/sj.bjc.6601442

**Published:** 2004-01-06

**Authors:** M P Laderoute

**Affiliations:** 1 Blood Zoonotics Unit, Blood Safety Surveillance and Health Care Acquired Infections Division, Centre for Infectious Disease Prevention and Control, Population and Public Health Branch, Health Canada, Tunney's Pasture AL 0601E2, Ottawa, Ontario Canada K1A 0L2

**Sir**,

The recent review article by Kneeshaw *et al* (2003) in the *British Journal of Cancer* regarding advances made in breast imaging by magnetic resonance is very encouraging and its expedited entry into routine clinical practice should be supported. This is not only due to its anticipated enhanced sensitivity and usefulness, but potentially also for its safety, particularly as may apply to radiation hypersensitivities such as female carriers of ataxia telangiectasia (AT).

Ataxia telangiectasia is a genetic cancer predisposition syndrome involving the overexpression of alpha-fetoprotein, immunosuppression of the host, advanced ageing, and a radiation hypersensitivity where AT homozygotes generally succumb to infection or to lymphoma before reaching adulthood (reviewed in Becker-Catania and Gatti, 2001). Female AT heterozygotes are at an increased risk of breast cancer, but this is usually detected after the age of 40 years (Swift *et al*, 1991; Swift, 2001,). The issue of safety of ionising radiation used for breast cancer screening has been previously raised for AT heterozygotes (Swift *et al*, 1991). About 1% of the population carries the genetic marker for AT, and 8–10% of all breast cancers appear to be AT heterozygotes (reviewed in Swift, 2001). This indicates the use of nonionising modes of breast imaging could make a significant impact on the incidence of breast cancers. However, other evidence suggests these potential benefits might also be extended into the general female population.

**Figure 1:**
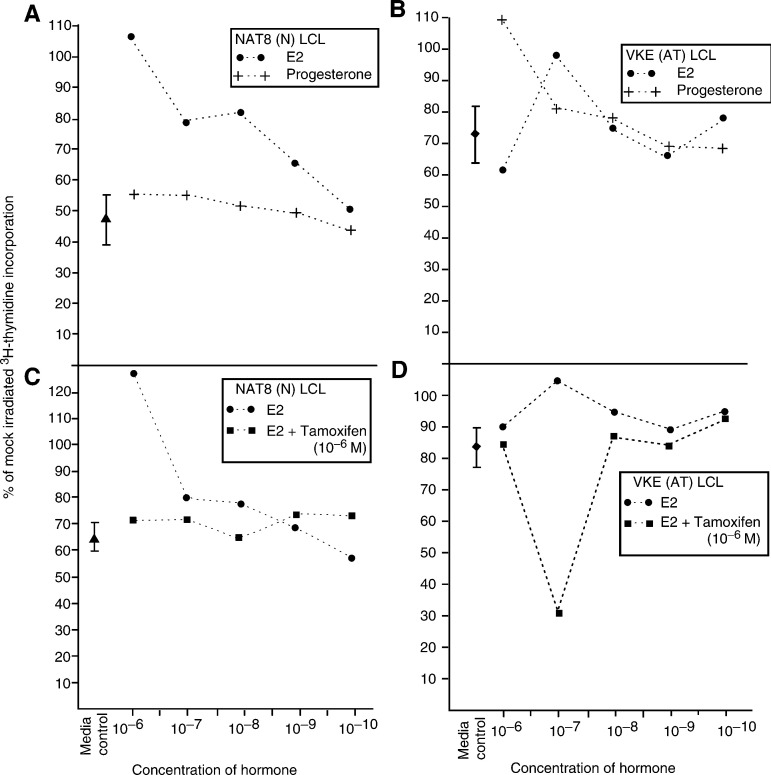
Estrogen specifically induces RDS in cells from normal individuals. Radioresistant DNA synthesis (RDS) measures the relative failure of cells from AT individuals to inhibit DNA synthesis at 1 h following ionising radiation exposures when compared to responses in normal cells that are conducted in parallel. To demonstrate this, the thymidine incorporation following ionising radiation is expressed as a percentage of that found when the cells are mock irradiated (see Laderoute (1996) for more details on methods). Panels (**A**) and (**B**) were conducted in parallel and as can be seen, the AT LCL labelled VKE show about 70% of the rate of DNA synthesis when the cells are not irradiated representing a 30% inhibition (see left side of panel **B**). In panel **A**, the LCLs labelled NAT8 derived from a normal individual has a postirradiation rate of about 45% of the level of DNA synthesis (left side of panel **A**) representing an inhibition of about 55%. The difference between the two (55%−30%=25%) represents RDS. As also shown in panel **A**, when the LCLs from normal individuals are treated overnight with oestrogen but not progesterone before performing the RDS assay, there is a reduced ability to inhibit DNA synthesis following irradiation, and this effect is proportional to the concentrations of oestrogen, implying a dose response. In panel **B**, we see in AT LCLs that RDS is relatively independent of added hormone. In the bottom panels (**C** and **D**), also conducted in parallel, it is that shown an excess of 10^−^6 M of tamoxifen added to the cells before estradiol is added, blocks the induction of RDS in LCLs from normals (panel **C**). In AT LCLs (panels **B** and **D**) tamoxifen or estrogen does not appreciably affect RDS except that at 10^−^7 M of oestrogen there seems to be some competition by tamoxifen. This may be consistent with the notion that genes implicated in RDS may have oestrogen response elements and which is also implicated by the results given in panels **A** and **C**. The dose rate was 68.9 cGy min^−1^ and cells were exposed to 2 Gy. The methods have been detailed earlier (Laderoute, 1996). Steroids were purchased from Sigma and stock solutions (10^−^2 M) were made in 70% ethanol immediately prior to dilution for addition to phenol red-free medium. Estradiol (E2) is an active version of oestrogen commonly used for *in vitro* experiments. Results are the means of triplicates and variance did not exceed 10%. The results are representative of three similar experiments. (Reproduced by kind permission of University of Ottawa Press from Laderoute, 1998).

A number of years ago while investigating the molecular biology of the AT radiation hypersensitivity, it was discovered that radioresistant DNA synthesis (RDS), the molecular signature of the radiation hypersensitivity of AT, could be modulated (Mirzayans *et al*, 1995; Mirzayans and Paterson, 2001). When c-myc and alpha-fetoprotein became implicated in the RDS phenotype (Laderoute, unpublished findings), oestrogen was tested for its ability to induce RDS in cells derived from normal individuals. As shown in Figure 1

, RDS was induced by estradiol (E2) but not progesterone in a dose-responsive fashion in lymphoblastoid cell lines (LCLs) derived from normal individuals (panel A). The induction was specific to oestrogen as it was blocked in the presence of an excess of tamoxifen (panel C), an oestrogen antagonist (Laderoute, 1998). Furthermore, additional experiments showed the RDS assay could be geared to easily detect low dose ionising radiation exposures around 1 rad or less in radiation hypersensitive cells such as those from AT individuals or AT heterozygotes (Laderoute, 1998). These results raised the possibility that breast imaging techniques using low-dose ionising radiation may induce breast cancers or cause existing breast tumours to progress, if the circulating levels of oestrogen are elevated in women at the time of mammography exposure. This phenomenon might help explain the reduced benefits in the under 50 years age-at-entry group reported in mammogram clinical trials (Miller *et al*, 1992, 2002), the increased incidence of breast cancer with mammogram screening among 45- to 64- year olds not accountable by earlier detection (White *et al*, 1990), and the lack of impact of mammography screening for reducing breast-cancer mortality overall (Gotzsche and Olsen, 2000).

It remains to be seen if the incidence of breast cancer returns to the premammogram screening rates of the 1960 s and 1970 s, as ionising radiation-based breast imaging technologies are replaced by MR imaging and as women decline hormone replacement therapies. One might also expect survival to again correlate inversely with tumour size, which is why early breast cancer imaging methods were introduced in the first place.
